# *In vitro* characterisation of Spontaneous Mammary Tumour (SMT1) cells and its matched lung metastatic (SMT1L) cells

**DOI:** 10.1186/1753-6561-6-S4-P17

**Published:** 2012-07-09

**Authors:** TC Wong, Y Smith, JH Harmey

**Affiliations:** 1 School of Biomolecular and Biomedical Sciences, University College Dublin, Ireland; 2 Molecular and Cellular Therapeutics, Royal College of Surgeons in Ireland, Ireland

## Introduction

Syngeneic mouse models of breast cancer offer the researcher an important tool with which to investigate the molecular mechanisms of cancer. These models generally arise through the isolation of breast cancer cells from spontaneous breast tumours in mice. As the cells have originated from a spontaneous tumour, they are immunologically and genetically compatible to a more clinically relevant breast cancer model. The aim of this study was to characterise newly isolated Spontaneous Mammary Tumour (SMT1) cells and its matched lung metastatic cells (SMT1L) from a spontaneous mammary tumour that arose in a 2 year old female BALB/c mouse.

## Methods

SMT1 and SMT1L cells were cultured in DMEM High Glucose supplemented with 10% FBS. Western blot analysis using cell lysates from SMT cells was used to confirm that cells were of epithelial origin, to characterise the cells for biomarkers of breast cancer subtypes: oestrogen receptor , progesterone receptor, ErbB2 (Her2) and Epidermal Growth Factor Receptor (EGFR) expression and to verify expression of the Insulin-like Growth Factor (IGF) pathway (the IGF receptor (IGF-IR) and Insulin Receptor (IR)). RT-PCR was used to confirm if SMT cells express Pregnancy-Associated Plasma Protein A (PAPP-A), a regulator of IGFBP4. The growth rates and effect of IGF-1 on growth of both SMT cell lines were compared by SRB proliferation assay.

## Results

SMT1 and SMT1L cells are of epithelial origin. SMT cell types expressed HER2, EGFR, and PR suggesting a more aggressive phenotype. The lack of ER positivity confirms this is not the main pathway of signalling in SMT cells. SMT cells express features of the IGF pathway including IR-β, IGF-1Rβ and PAPP-A. SMT1 and SMT1L proliferation rates *in vitro* were similar, with both cell lines responding to IGF-1 stimulation showing increased growth compared to untreated controls.

## Conclusions

Based on expression of common biomarkers of breast cancer subtypes, SMT cells may be more aggressive cell line. Expression of members of the IGF pathway and response to IGF-I stimulation indicates that inhibiting IGF may be of value in inhibiting growth of SMT tumours, and It may offer another preclinical syngeneic model of breast cancer.

